# NSC 95397 Suppresses Proliferation and Induces Apoptosis in Colon Cancer Cells through MKP-1 and the ERK1/2 Pathway

**DOI:** 10.3390/ijms19061625

**Published:** 2018-05-31

**Authors:** Navneet Kumar Dubey, Bou-Yue Peng, Chien-Min Lin, Peter D. Wang, Joseph R. Wang, Chun-Hao Chan, Hong-Jian Wei, Win-Ping Deng

**Affiliations:** 1 Ceramics and Biomaterials Research Group, Advanced Institute of Materials Science, Ton Duc Thang University, Ho Chi Minh 700000, Vietnam; navneet.kumar.dubey@tdt.edu.vn; 2 Faculty of Applied Sciences, Ton Duc Thang University, Ho Chi Minh 700000, Vietnam; 3 School of Dentistry, College of Oral Medicine, Taipei Medical University, Taipei 110, Taiwan; pemg@tmu.edu.tw (B.-Y.P.); dpw1@tmu.edu.tw (P.D.W.); harry811003@gmail.com (C.-H.C.); 4 Department of Dentistry, Taipei Medical University Hospital, Taipei 110, Taiwan; 5 Department of Neurosurgery, Taipei Medical University–Shuang Ho Hospital, Ministry of Health and Welfare, New Taipei 235, Taiwan; m513092004@tmu.edu.tw; 6 Department of Periodontics, College of Dental Medicine, Columbia University, New York, NY 10032, USA; jrw2166@cumc.columbia.edu; 7 Stem Cell Research Center, Taipei Medical University, Taipei 110, Taiwan; 8 School of Dental Technology, College of Oral Medicine, Taipei Medical University, Taipei 110, Taiwan

**Keywords:** NSC 95397, MKP-1, ERK1/2, colon cancer, antiproliferation, apoptosis

## Abstract

NSC 95397, a quinone-based small molecule compound, has been identified as an inhibitor for dual-specificity phosphatases, including mitogen-activated protein kinase phosphatase-1 (MKP-1). MKP-1 is known to inactivate mitogen-activated protein kinases by dephosphorylating both of their threonine and tyrosine residues. Moreover, owing to their participation in tumorigenesis and drug resistance in colon cancer cells, MKP-1 is an attractive therapeutic target for colon cancer treatment. We therefore investigated the inhibitory activity of NSC 95397 against three colon cancer cell lines including SW480, SW620, and DLD-1, and their underlying mechanisms. The results demonstrated that NSC 95397 reduced cell viability and anchorage-independent growth of all the three colon cancer cell lines through inhibited proliferation and induced apoptosis via regulating cell-cycle-related proteins, including p21, cyclin-dependent kinases, and caspases. Besides, by using mitogen-activated protein kinase kinase (MEK)/extracellular signal-regulated kinase (ERK) inhibitor U0126, we provided mechanistic evidence that the antineoplastic effects of NSC 95397 were achieved via inhibiting MKP-1 activity followed by ERK1/2 phosphorylation. Conclusively, our results indicated that NSC 95397 might serve as an effective therapeutic intervention for colon cancer through regulating MKP-1 and ERK1/2 pathway.

## 1. Introduction

Mitogen-activated protein kinase (MAPK) pathways play important roles in a broad spectrum of cellular processes such as cell proliferation, differentiation, migration and apoptosis. MAPKs are a group of highly conserved protein kinases comprising several subclasses, including extracellular signal-regulated protein kinases 1 and 2 (ERK1/2), c-Jun N-terminal kinases (JNKs), and p38 MAPKs, and mediate both physiological and pathological responses to various extracellular and intracellular stimuli. MAPK signaling cascades are activated by sequential phosphorylation events and negatively regulated by dual-specificity MAPK phosphatases [1]. Mitogen-activated protein kinase phosphatase-1 (MKP-1), a member of dual-specificity phosphatases, inactivates MAPKs by dephosphorylating both their threonine and tyrosine residues and has been correlated with tumor progression in many cancers during its altered expression [2]. Overexpression of MKP-1 has been detected in several cancers, including breast [3], lung [4], prostate [5,6], ovarian [7], pancreatic [8], and gastric cancer [9]. On the contrary, MKP-1 expression was decreased in liver [10,11] and head and neck cancers [12]. MKP-1 has also been shown to promote angiogenesis and metastasis in lung cancer [13]; but inhibit cell proliferation, metastasis, and angiogenesis in gallbladder cancer [14]. Thus, MKP-1 seemed to play either pro- or anti-tumor role depending on the specific tumor context. However, accumulated evidences show that MKP-1 is involved in resistance to anticancer treatment, and confer cisplatin resistance in many cancers [15,16,17]. Besides, the inhibition of MKP-1 also promoted gemcitabine sensitivity in pancreatic cancer [18], dexamethasone sensitivity in lung cancer [19], and the sensitivities of three chemotherapy drugs (mechlorethamine, doxorubicin, and paclitaxel) in breast cancer [20]. Although, the mechanisms by which MKP-1 reduces drug sensitivity in cancer have not been fully elucidated, the decreased MAPKs-induced apoptosis has been implicated in MKP-1-activated chemoresistance.

Colon cancer is one of the most common cancers worldwide, causing approximately 1.4 million incidences and 693,900 deaths in 2012 [21]. Notably, in colon cancer, MKP-1 was overexpressed in the early phases of carcinogenesis [22] and impaired the response to cetuximab-based treatment in metastatic colon cancer patients [23]. Furthermore, the therapeutic effect of camptothecin in colon cancer cells was improved through inhibition of MKP1 activity [24]. These studies suggest that MKP-1 may be an attractive target for colon cancer therapy. NSC 95397, a cell-permeable para-naphthoquinone, was initially identified as a selective inhibitor of Cdc25 dual specificity phosphatases [25]. Due to the involvement of Cdc25 phosphatases in cell cycle progression, NSC 95397 was first examined for anti-proliferative potential; and reduced the growth of several cancer cells [26,27,28,29]. In addition to Cdc25 phosphatase, MKP-1 has been demonstrated as a primary target for the inhibitory activity of NSC 95397 [30]. Although the antineoplastic potential of NSC 95397 has been revealed, the underlying mechanisms by which NSC 95397 suppresses colon cancer cells have not been fully elucidated. Therefore, in this study, we aimed to investigate the antitumor activity of NSC 95397 on colon cancer cells and their underlying mechanisms. Our results demonstrated that NSC 95397 reduces cell proliferation and induces apoptosis in colon cancer cells. Specifically, the cyclin-dependent kinase inhibitor p21 and activated apoptotic protein caspase-3 were induced by NSC 95397 via inhibition of MKP-1 activity and induction of ERK1/2 phosphorylation. Collectively, the data suggest that the antineoplastic effects of NSC 95397 on colon cancer cells might be achieved by regulating MKP-1/ERK1/2 pathway.

## 2. Results

### 2.1. NSC 95397 Reduces Cell Viability of Colon Cancer Cells

**Figure 1:**
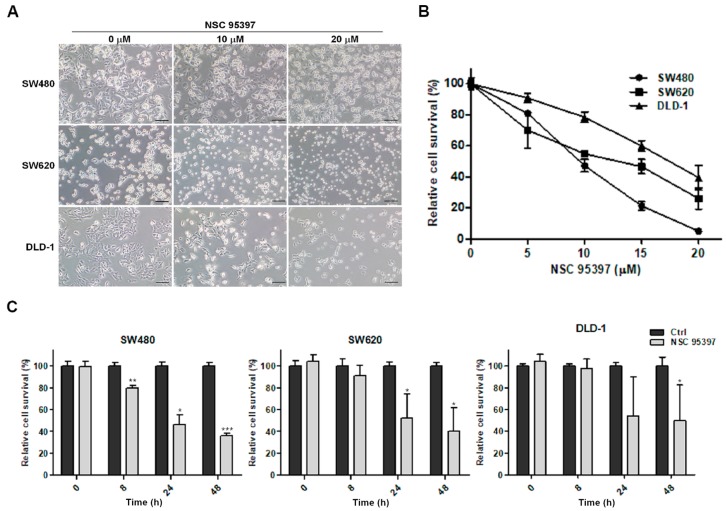
Inhibitory effect of NSC 95397 on cell viability in human colon cancer cells. (**A**) Morphological changes of SW480, SW620, and DLD-1 cells after exposure to NSC 95397 for 24 h. Scale bars represent 100 μm; (**B**) Cell viability (mean ± SD) of SW480, SW620, and DLD-1 cells treated with indicated concentrations of NSC 95397 for 24 h assessed by MTT assay; (**C**) Cell viability of SW480, SW620, and DLD-1 cells treated with 10 μM NSC 95397 for indicated times assessed by MTT assay. Values are means + SD; * *p* < 0.05; ** *p* < 0.01; and *** *p* < 0.001 using unpaired *t*-tests with Welch’s correction, compared to relative control group.

To evaluate the toxicity of exposed NSC 95397 on three human colon cancer cell lines, SW480, SW620, and DLD-1, the changes in cellular morphology was observed and MTT assay were conducted to assess cellular viability. After treatment of NSC 95397 for 24 h, the cellular morphology of all the cells was markedly changed with the increasing dosages (0, 10, and 20 µM), and revealed a reduced cell density, cell shrinkage, and irregular shape (Figure 1A). Change of cellular morphology was directly related to cell viability. Further, these degenerative changes were corroborated through MTT assay. We found that NSC 95397 decreases the cell viability of three colon cancer cell lines in a concentration-dependent manner (Figure 1B). Specifically, the IC_50_ values of NSC 95397 for the cell growth of SW480, SW620, and DLD-1 cells were 9.9, 14.1 and 18.6 μM, respectively, indicating that SW480 cells appeared to be more sensitive than SW620 and DLD-1 cells. As shown in Figure 1C, 10 μM NSC 95397 revealed an inhibitory effect on SW480 cells but showed no significant change on SW620 and DLD-1 cells at 8 h. As time progressed, the significantly reduced viability of SW480, SW620, and DLD-1 cells was found at 24 h, which were maintained until 48 h. These results showed that NSC 95397 inhibited the cell viability of SW480, SW620, and DLD-1 in a dose- and time-dependent manner. 

### 2.2. NSC 95397 Suppresses Soft Agar Colony Formation in Colon Cancer Cells

**Figure 2:**
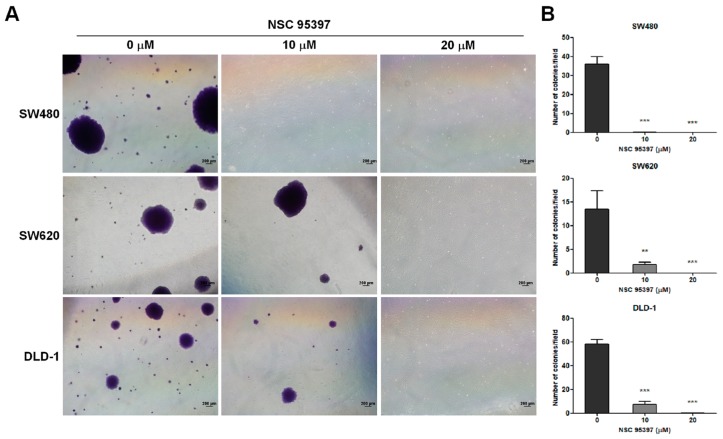
NSC 95397 reduces anchorage independent growth of human colon cancer cells. (**A**) Representative phase-contrast images and (**B**) quantitation of colony formed by SW480, SW620, and DLD-1 cells treated with indicated concentrations of NSC 95397; scale bars indicate 200 μm. Values are means + SD; ** *p* < 0.01; and *** *p* < 0.001 using one-way analysis of variance (ANOVA), compared to vehicle control (0 μM).

As anchorage-independent growth is a hallmark of carcinogenesis, we evaluated the impact of NSC 95397 on the clonogenic ability of colon cancer cells in soft agar. Representative images are shown in Figure 2A, which were further quantified as shown in Figure 2B. In the presence of 10 μM NSC 95397, colony formation of SW620 and DLD-1 cells was significantly decreased in soft agar, whereas the clonogenicity of SW480 cells was almost diminished. Moreover, all three cell lines could not form any colonies upon treatment with 20 μM NSC 95397. The results indicated that NSC 95397 suppressed the capacity of anchorage-independent growth in colon cancer cells.

### 2.3. NSC 95397 Reduces Cell Proliferation by Inhibiting the Expression of Cell Cycle Regulatory Proteins

**Figure 3:**
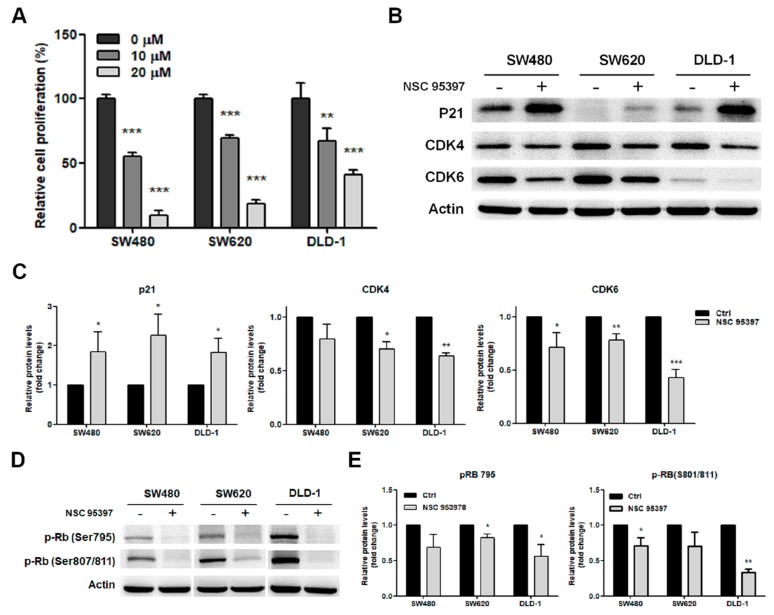
Inhibitory effect of NSC 95397 on cell proliferation and expression of cell cycle regulatory proteins. (**A**) In vitro cell proliferation (mean + SD) of SW480, SW620, and DLD-1 cells treated with indicated concentrations of NSC 95397 for 24 h assessed by BrdU assay; ** *p* < 0.01; and *** *p* < 0.001 using one-way ANOVA, compared to vehicle control (0 μM); (**B**) Representative Western blots showing expression of CDK4, CDK6, and p21 in SW480, SW620, and DLD-1 cells treated with 10 μM NSC 95397 for 24 h, with actin as loading control; (**C**) Quantitative analysis of the relative protein expression of p21, CDK4, and CDK6 normalized actin. Values (means + SD) are normalized to actin loading control; * *p* < 0.05; ** *p* < 0.01; and *** *p* < 0.001 using paired *t*-tests, compared to control group; (**D**) Representative Western blots showing of the phosphorylation of Rb at Ser795 and Ser807/811 in SW480, SW620, and DLD-1 cells treated with 10 μM NSC 95397 for 24 h, with actin as loading control; (**E**) Quantitative analysis of the relative protein phosphorylation of Rb at Ser795 and Ser807/811 normalized actin. Values (means + SD) are normalized to actin loading control; * *p* < 0.05; and ** *p* < 0.01 using paired *t*-tests, compared to control group.

To identify whether NSC 95397 reduces cell proliferation, we measured bromodeoxyuridine (BrdU) incorporation in colon cancer cells treated with NSC 95397. After 24-h treatment, BrdU incorporation was significantly reduced in SW480, SW620, and DLD-1 cells by 10 and 20 μM NSC 95397 in a concentration-dependent manner (Figure 3A). SW480 cells appeared to be most sensitive among these three cell lines, which is in agreement with reduced cell viability results (Figure 1). The changes in cell proliferation suggested that NSC 95397 might affect the expression pattern of cell cycle proteins. Therefore, we further explored this possibility by measuring levels of cell cycle regulatory proteins by Western blot. The results revealed that, upon NSC 95397 treatment, p21 was upregulated while cyclin-dependent kinases (CDKs) 4 and 6 were downregulated in all three colon cancer cell lines (Figure 3B,C). CDK4 and CDK6 are master integrators that couple mitogenic and oncogenic signals with the phosphorylation and inactivation of the tumor suppressor retinoblastoma protein (Rb). Furthermore, p21 can inhibit the activity of cyclin-CDK2 and -CDK4/6 complexes that lead to dephosphorylation and the activation of Rb [31]. Hence, we further evaluated the levels of Rb phosphorylation and found that NSC 95397 reduced the phosphorylation of Rb on Ser795 and Ser807/811 in colon cancer cells (Figure 3D,E). However, after NSC 95397 treatment, a smaller decrease of pRb was exhibited in SW620 cells compared to SW480 and DLD-1 cells. The weaker inhibitory effect of NSC 95397 on Rb phosphorylation might result due to low levels of p21 in SW620 cells. Collectively, NSC 95397 treatment promotes p21 expression, reduces CDK4/6 expression and Rb phosphorylation, and thus suppresses the proliferation of colon cancer cells.

### 2.4. NSC 95397 Induces Apoptosis in Colon Cancer Cells

**Figure 4:**
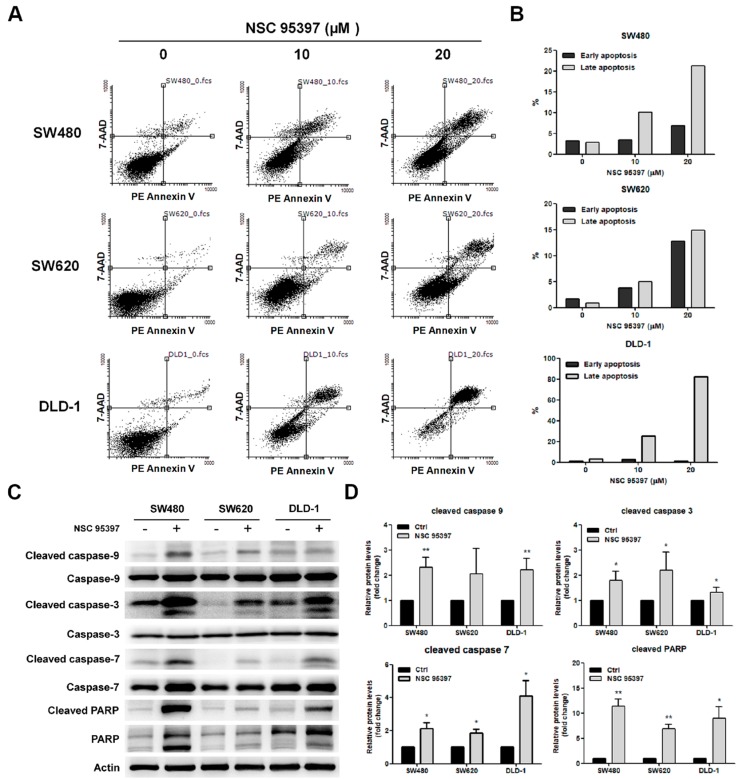
Apoptosis induction of NSC 95397 in human colon cancer. (**A**) Flow cytometric analysis of Annexin V/7-AAD staining in SW480, SW620, and DLD-1 cells treated with indicated concentrations of NSC 95397 for 24 h. Annexin V and 7-AAD negative were designated as live cells in the lower left quadrant; Annexin V positive and 7-AAD negative as early apoptotic cells in the lower right quadrant; Annexin V positive and 7-AAD positive as late apoptotic cells in the upper right quadrant. (**B**) Quantitative analysis of Annexin V/7-AAD staining; (**C**) representative Western blots showing expression of cleaved caspase-9, caspase-9, cleaved caspase-3, caspase-3, cleaved caspase-7, caspase-7, cleaved PARP, and PARP in SW480, SW620, and DLD-1 cells treated with 20 μM NSC 95397 for 24 h, with actin as loading control. (**D**) Quantitative analysis of the relative protein expression of cleaved caspase-9, cleaved caspase-3, cleaved caspase-7, and cleaved PARP normalized actin. Values (means + SD) are normalized to actin loading control; * *p* < 0.05; and ** *p* < 0.01 using paired *t*-tests.

Since a significant inhibitory effect of NSC 95397 on colon cancer cells was observed, we then assessed whether NSC 95397 induced apoptosis in colon cancer cells. The cell apoptosis was evaluated by Annexin V/7-aminoactinomycin D (7-AAD) staining. Annexin V can be detected in both early and late stages of apoptosis, whereas 7-AAD enters cells in late apoptosis or necrosis. As shown in Figure 4A, apoptotic SW480, SW620, and DLD-1 cells in lower right quadrant (early apoptosis) and upper right quadrant (late apoptosis) were markedly increased with NSC 95397 concentration. The quantitative results showed that the percentage of early and late apoptotic cells increased gradually with NSC 95397 concentration in SW480 and SW620 cells, while DLD-1 cells underwent a dramatic late apoptosis following NSC 95397 treatment (Figure 4B). Moreover, the activation of the caspase cascade is thought to be a hallmark of apoptosis [32]. Thus, we conducted Western blot assay to assess caspase activation. We found that NSC 95397 significantly increased cleaved caspase-9, -3, -7 and poly(ADP-ribose) polymerase (PARP) levels in SW480, SW620, and DLD-1 cells (Figure 4C). These results were further confirmed through quantification of expressed protein (Figure 4D). Overall, these results suggest that NSC 95397 induces colon cancer cell death through activation of a caspase-dependent apoptotic mechanism.

### 2.5. NSC 95397 Enhances p21 Expression and Caspase-3 Activity via ERK1/2 Activation

**Figure 5:**
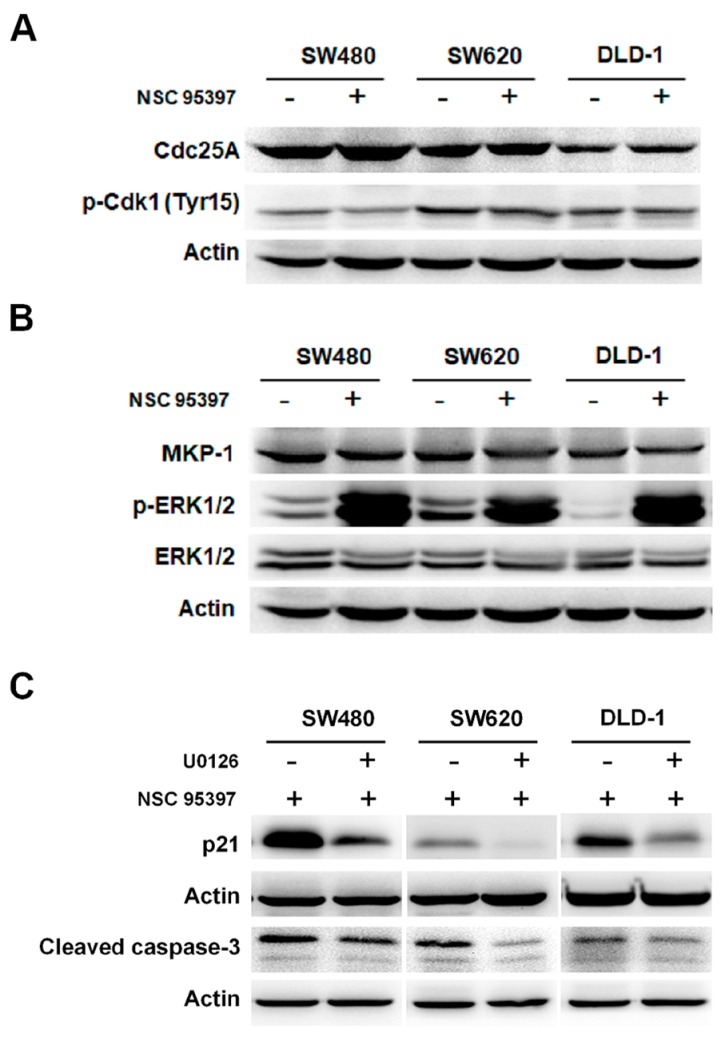
Involvement of MKP-1/ERK1/2 pathway in NSC 95397-induced p21 expression and caspase-3 activation in human colon cancer. Representative Western blots showing (**A**) the expression of Cdc25A, the phosphorylation of Cdk1 at Tyr15; (**B**) the expression of MKP-1 and ERK1/2, and the phosphorylation of ERK1/2 at Thr202/Tyr 204 in SW480, SW620, and DLD-1 cells treated with 10 μM NSC 95397 for 6 h, with actin as loading control; (**C**) representative Western blots showing expression of p21 and cleaved caspase-3 in colon cancer cells that were pre-incubated with 20 μM U0126 for 2 h and then treated with 10 m μM NSC 95397 for 6 h.

Dual-specificity phosphatases (DUSPs) are a superfamily of protein phosphatases that can dephosphorylate both tyrosine and serine/threonine residues. DUSPs have been implicated as critical regulators of malignant characteristics in various cancers [33]. NSC 95397 is a selective and effective quinone-based, dual-specificity phosphatase inhibitor that has been reported to inhibit Cdc25 [25] and MKP-1 [30]. To further investigate the mechanisms involved in the inhibitory effect of NSC 95397 on colon cancer cells, we evaluated whether NSC 95397 inhibits Cdc25A and/or MKP-1 in colon cancer cells. First, we found that NSC 95397 does not reduce the protein level of Cdc25A and the dephosphorylation of downstream protein Cdk1 in colon cancer cells (Figure 5A). However, though NSC 95397 did not decrease MKP-1 expression, it enhanced the phosphorylation of its downstream ERK1/2 (Figure 5B). This result corresponds to the previous report showing NSC 95397 inhibited MKP-1 activity rather than expression levels [30]. Further, as aforementioned results indicate that NSC 95397 upregulates the expression of p21, a master regulator of multiple tumor suppressor pathways in colon cancer cells, we utilized specific MEK/ERK inhibitor U0126 to investigate whether NSC 95397 induced p21 expression via ERK1/2 activation. As mentioned before that protein levels of p21 are upregulated in SW480, SW620, and DLD-1 cells upon NSC 95397 treatment (Figure 3B), whereas U0126 markedly reduced the NSC 95397-induced upregulation of p21 expression in these cells (Figure 5C). Moreover, similar results were found for caspase-3 activity that the NSC 95397-induced upregulation of cleaved caspase-3 in colon cancer cells was suppressed by U0126 (Figure 5C). Collectively, NSC 95397 increased the expression of p21 and cleaved caspase-3 in an ERK1/2-dependent manner, indicating that the NSC 95397-induced inhibitory activities, including anti-proliferation and pro-apoptosis, on colon cancer cells might be through inhibition of MKP-1 activity and induction of ERK1/2 phosphorylation.

## 3. Discussion

NSC 95397 is a quinone-based small molecule compound that was first evaluated as an inhibitor for Cdc25 dual-specificity phosphatases [25]. Because of the importance of Cdc25 in cell cycle control, NSC 95397 has been assessed for its anti-proliferative potential in several cancers [26,27]. However, in the present study, we revealed the anti-proliferative and pro-apoptotic effects of NSC 95397 on colon cancer cells independently of Cdc25. We provided mechanistic evidence that the antineoplastic effects of NSC 95397 were achieved through MKP-1/ERK1/2 pathway. We found that NSC 95397 reduced cell viability and anchorage-independent growth capacity in SW480, SW620, and DLD-1 colon cancer cells and SW480 cells appears to be most sensitive among these three colon cancer cell lines. However, the underlying mechanisms of drug response in cancer cells are extremely complex. For example, cancer cells can develop drug resistance through drug target alteration, drug inactivation, drug efflux, DNA mutations, metabolic changes, and apoptotic inhibition [34]. Although the exact cause of behind highest sensitivity of SW480 towards NSC 95397 remains unclear; interestingly we observed the varying expressions profile of MKP-1 in SW480, SW620, and DLD-1 cells. We utilized real-time PCR and Western blot analyses to evaluate mRNA and protein levels of MKP-1 in SW480, SW620, and DLD-1 cells. We found that the MKP-1 expression was higher in SW480 cells than in SW620 and DLD-1 cells (Figure S1). Thus, the highest sensitivity of SW480 cells to NSC 95397 may be attributed to higher MKP-1 expression.

Here, we showed that NSC 95397 upregulates the expression of p21 in SW480, SW620, and DLD-1 cells. The cyclin-dependent kinase inhibitor p21, a p53-downstream target, facilitates p53-dependent cell cycle arrest in response to various stimuli [35]. Thus, deregulated p21 expression is involved in many pathological symptoms such as carcinogenesis, senescence, and age-related diseases. However, all three colon cancer cell lines used in this study contain p53 mutations. SW480 and SW620 cells are derived from the same individual with a similar p53 alteration (containing mutant p53 R273H/P309S) [36,37,38], while DLD-1 cells contain mutant p53-S241F [39]. Many studies have identified that p21 plays a crucial role in the various pathways of tumor suppression for inhibiting cell proliferation through p53-independent pathway [40,41]. Thus, our results suggested that NSC 95397-induced upregulation of p21 expression in colon cancer cells might be mediated through p53-independent manner. Furthermore, accumulating evidence shows that p21 inhibits cancer cell growth by inducing cell cycle arrest or apoptosis when cancer cells expose to therapeutic agents. Increased expression of p21 by therapeutic agents has been observed in various cancers such as lung [42], liver [43], ovarian [44], and colon cancers [45]. p21 can inhibit the formation of CDK/cyclin complexes, thereby decreasing the phosphorylation of Rb. In addition to p21, NSC 95397 also reduced the expression of CDK4 and CDK6 proteins, largely involved in cell cycle progression. Collectively, NSC 95397-induced cell growth inhibition was simultaneously orchestrated by over-expression of p21 and downregulated expression of CDK4 and CDK6.

In addition to anti-proliferation, we also found that NSC 95397 could induce apoptosis in colon cancer cells. Programmed cell death by apoptosis is a universal and effective cellular suicidal pathway, since it is required in various biological processes, such as embryogenesis, tissue homeostasis, immunity, and aging [46]. Apoptosis is caused by caspases that are a family of intracellular cysteine aspartyl-specific proteases. During apoptosis, activation of caspases ensures that the cellular components are degraded in a controlled mode, leading to cell death without affecting the surrounding tissues. Caspases are the central components of the apoptotic response and generally divided into two classes: the initiator caspases, including caspase-2, 8, 9 and 10 and the effector caspases, including caspases-3, 6 and 7 [47]. All caspases are produced in cells as catalytically inactive zymogens and need to undergo an activation process. Once activated, these caspases cleave a broad spectrum of cellular components that are required for normal cellular function, which ultimately leads to cell death [32]. Although several reports have shown the anti-proliferative property of NSC 95397 on cancer cells, the pro-apoptotic effect of NSC 95397 has only been identified by Larsson et al., on neuroendocrine tumors [29]. Herein, we revealed that NSC 95397 induces the apoptosis of colon cancer cells via activation of caspase-9, 3, 7 and PARP.

As aforementioned, NSC 95397 is a quinone-based small molecule compound, which has been demonstrated its growth-inhibitory effect in several studies at concentrations of 5–20 µM [25,26,27,29,30,48]. These studies also showed that the inhibitory target of NSC 95397 is either Cdc25 or MKP-1. Although the study by Yang et al. revealed that NSC 95397 exerts inhibitory effect on multiple kinases such as AKT, IκB kinase (IKK) α/β, mitogen-activated protein kinase kinase 7 (MKK7), and TANK-binding kinase 1 (TBK1), the reported working concentration of NSC 95397 was up to 40 µM [49]. However, in most of our experiments, 10 µM of NSC 95397 showed significant inhibitory activity on colon cancer cells without inhibiting the protein levels of Cdc25A and the Tyr-15 dephosphorylation of Cdk1. In addition to Cdc25A, Cdc25B and Cdc25C, the other two members of Cdc25 family, can also dephosphorylated Cdk1 at Tyr-15 [50]. Therefore, our results suggested that Cdc25 might not contribute to the antineoplastic effects of NSC 95397 on colon cancer cells in the present study. Furthermore, corresponding to previous report [30], NSC 95397 suppressed MKP-1 activity rather than expression levels and enhanced the phosphorylation of its downstream ERK1/2 in our study. On the other hand, quinones are known to readily undergo either one-electron reduction to form semiquinone or two-electron reduction to form hydroquinone. Semiquinone can then produce additional electron to oxygen and form superoxide radicals and enhance oxidative stress. Hydroquinone is relatively stable and involved in the detoxification pathway of cancer cells. These one or two-electron reduction of quinones is catalyzed by nicotinamide adenine dinucleotide phosphate (NADPH)-cytochrome P450 reductase and NADPH-quinone oxidoreductase-1 (NQO1), respectively [51]. However, Han et al. documented that NSC 95397 is reduced by NQO1 instead of one-electron reducing enzymes. They further identified that NSC 95397-generated cytotoxicity is not affected by NQO1, suggesting that the cytotoxic effects of NSC 95397 were not mainly achieved by oxidative stress [27]. Hence, we evaluated the mRNA expression of NQO1 in SW480, SW620, and DLD-1 cells by real-time PCR analysis and found no correlation between NQO1 expression and NSC 95397-induced cytotoxicity (Figure 1 and Figure S2). Moreover, Vogt et al. have identified that the generation of ROS is not sufficient to inhibit cellular MKP-1; therefore, it seems unlikely that ROS are the main cause for cellular MKP1 inhibition by NSC 95397 [30].

MAPK signaling pathways play a crucial role in almost all cellular functions and are frequently deregulated in human cancers [1]. It is well-documented that MAPK signaling pathways promote apoptosis during various conditions of stress. The ERK signaling is the best studied of MAPK pathways, and generally activated in response to survival signal that counteracts apoptotic stimuli from JNK and p38 activation [52]. Nonetheless, ERK signaling has also been identified to promote different anti-proliferative properties, including apoptosis, autophagy, and senescence [53]. Interestingly, upon using MEK/ERK inhibitor U0126, we found that NSC 95397-induced p21 upregulation is activated via MKP-1/ERK1/2 proteins. As above-mentioned, p21 is an important tumor suppressor that promotes cell cycle arrest and cellular senescence. p21 has been demonstrated to promote cellular senescence upon the activation of ERK1/2 signaling in various cell types [53]. Moreover, ERK1/2 activity has been involved in p21-induced cell-cycle arrest caused by antitumor compounds [54,55]. On the other hand, ERK1/2 activation has been proved to facilitate cellular apoptosis induced by DNA-damaging stimuli such as chemotherapeutic agents, ultraviolet radiation and γ irradiation [53]. ERK1/2 activity has been implicated in cancer cell apoptosis induced by various antitumor compounds, including etoposide [56], doxorubicin [57], cisplatin [58], taxol [59], miltefosine [60], and shikonin [61]. Importantly, we found that the NSC 95397-activated caspase 3, which is responsible for cleavage of many nuclear proteins essential for apoptosis-associated DNA fragmentation, chromatin margination, and nuclear collapse, was suppressed by MEK/ERK inhibitor U0126. This result suggests that ERK1/2 activation is implicated in the NSC 95397-induced apoptosis of colon cancer cells. However, NSC 95397 has also been shown to induce cell death through other signalings such as the induction of erythrocyte death by Ca^2+^ entry and protein kinase C activation [62]. Although we could not exclude the possibility that NSC 95397 might have alternative or secondary pathways, such as p38 MAPK, oxidative stress, and other DUSPs, to induce cell apoptosis, the current results demonstrated that ERK1/2 activation contributes to the antineoplastic effects of NSC 95397, including anti-proliferation and apoptosis. 

## 4. Materials and Methods

### 4.1. Cell Culture

Three human colorectal adenocarcinoma cell lines SW480 (ATCC^®^ CCL-228™), SW620 (ATCC^®^ CCL-227™), and DLD-1 (ATCC^®^ CCL-221™) were cultured in RPMI-1640 medium supplemented with 10% fetal bovine serum (Thermo Fisher Scientific, Waltham, MA, USA) and 1% Antibiotic-Antimycotic (Thermo Fisher Scientific, Waltham, MA, USA) in a humidified atmosphere with 5% CO_2_ at 37 °C.

### 4.2. Cell Viability Assay

Cell viability was determined with MTT assay using Thiazolyl Blue Tetrazolium Bromide (Sigma-Aldrich, St. Louis, MO, USA). SW480 (1 × 10^4^), SW620 (2 × 10^4^), and DLD-1 (1 × 10^4^) cells were seeded into 96-well plates using eight wells/cell line/time point. After an overnight culture, cells were treated with the indicated time and concentration of NSC 95397. The MTT reagent was added into each well after NSC 95397 treatment. O.D. values (O.D. 595∼690) were analyzed 4 h after addition of MTT reagent using a Multiskan PC (Thermo Labsystem, Beverly, MA, USA)

### 4.3. Anchorage-Independent Growth

We added 1 mL of 0.6% agar in complete growth medium in each well of six-well plates as a base agar. Top agar was prepared by 1 mL of 0.35% agar in complete growth medium with 5 × 10^3^ cells and overlaid on the base agar. Growth medium (2 mL) containing indicated concentration of NSC 95397 was added on top of the second layer and changed twice a week. After incubation for four weeks, the forming colonies were stained with 10% crystal violet in methanol (Fisher Scientific, Hampton, NH, USA) and then we calculated their number.

### 4.4. Cell Proliferation Assay

To measure the cell proliferation activity of NSC 95397 against colon cancer cells, SW480 (1 × 10^4^), SW620 (2 × 10^4^), and DLD-1 (1 × 10^4^) cells were seeded into 96-well plates. After overnight culture, NSC 95397 cells were added at the indicated concentrations. After 24 h of incubation, cell proliferation was determined in vitro using a BrdU cell proliferation assay kit (Merck Millipore Burlington, MA, USA) according to the manufacturer’s instructions. O.D. 450 values were analyzed by using a Multiskan PC (Thermo Labsystem, Beverly, MA, USA).

### 4.5. Apoptosis Assay

The apoptosis of colon cancer cells was determined by a PE Annexin V Apoptosis Detection Kit with 7-AAD (BioLegend, San Diego, CA, USA) according to the manufacturer’s instructions. Briefly, cells were treated with the indicated concentration of NSC 95397 for 24 h. After 24 h, Cells were harvested and stained with PE Annexin V/7-AAD for 15 min. The stained cells were analyzed using FACSCanto II low cytometer (BD Biosciences, Franklin Lakes, NJ, USA) and FCS Express software (De Novo, Glendale, CA, USA).

### 4.6. Western Blot Analysis

The protein extraction and immunoblotting were performed as previously described [63]. The following antibodies were used: rabbit monoclonal anti-p21 (Cell Signaling Technology #2947, Danvers, MA, USA), rabbit monoclonal anti-CDK4 (Cell Signaling Technology #12790), mouse monoclonal anti-CDK6 (Cell Signaling Technology #3136), rabbit polyclonal anti-Phospho-Rb (Ser795) (Cell Signaling Technology #9301), rabbit monoclonal anti-Phospho-Rb (Ser807/811) (Cell Signaling Technology #8516), mouse monoclonal anti-Caspase-9 (Cell Signaling Technology #9608), rabbit monoclonal anti-Cleaved Caspase-9 (Cell Signaling Technology #7237), rabbit monoclonal anti-Caspase-3 (Cell Signaling Technology #9665), rabbit monoclonal anti-Cleaved Caspase-3 (Cell Signaling Technology #9664), rabbit monoclonal anti-Caspase-7 (Cell Signaling Technology #12827), rabbit monoclonal anti-Cleaved Caspase-7 (Cell Signaling Technology #8438), rabbit polyclonal anti-PARP (Cell Signaling Technology #9542), rabbit monoclonal anti-Cleaved PARP (Cell Signaling Technology #5625), rabbit polyclonal anti-cdc25A (Cell Signaling Technology #3652), rabbit polyclonal anti-Phospho-cdc2 (Tyr15) (Cell Signaling Technology #4539), rabbit polyclonal anti-MKP-1 (Bioworld Technology #BS1677, St. Louis Park, MN, USA), rabbit polyclonal anti-ERK1/2 (GeneTex #GTX59618, Irvine, CA, USA), rabbit polyclonal anti-Phospho-ERK1/2 (GeneTex #GTX61126), and mouse monoclonal anti-Actin (Merck Millipore #MAB1501, Burlington, MA, USA).

### 4.7. Statistical Analysis and Replicates

The sample size in each experiment is at least *n* = 3, unless otherwise indicated. All data are representative of at least three independent experiments that generated similar results. Statistical analyses were conducted by utilizing GraphPad Prism 5 (version 5.01, GraphPad Software, San Diego, CA, USA). 

## 5. Conclusions

**Figure 6:**
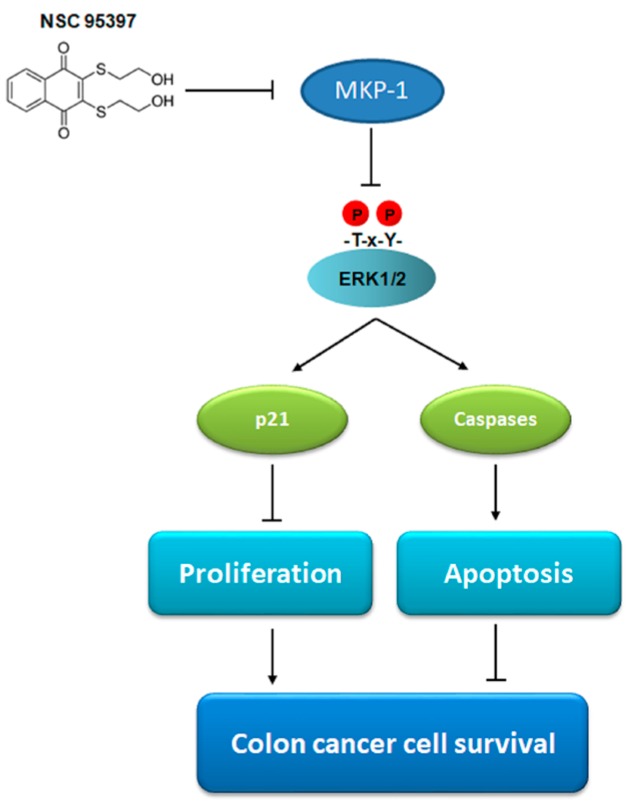
A schematic showing that the cytotoxic effect of NSC 95397 on colon cancer cells is activated by inhibiting MKP-1 activity followed by ERK1/2 activation. Lines ending in arrows indicate promotion and T-bars represent inhibition.

Taken together, we demonstrated that NSC 95397 reduces cell viability and anchorage-independent growth as well as induces apoptosis in colon cancer cells. The anti-proliferative and pro-apoptotic effects of NSC 95397 on colon cancer cells were achieved by regulating cell cycle proteins, including p21, CDKs, and caspases. Upon using MEK/ERK inhibitor U0126, it was demonstrated that the major proteins associated with proliferation and apoptosis, p21 and caspase-3, are activated in a MKP-1/ERK1/2-dependent manner. Conclusively, NSC 95397 exerts anti-proliferative and pro-apoptotic effects on colon cancer cells via inhibiting MKP-1 activity followed by ERK1/2 activation (Figure 6). Accumulating evidence has indicated that MKP-1 confers drug resistance in various cancers, suggesting that MKP-1 inhibition could enhance the efficiency of conventional chemotherapy. Furthermore, NSC 95397 has been used as a cell-active MKP-1 inhibitor to restore paclitaxel-induced apoptosis in resistant cancer cells [30]. Our overall findings, coupled with previous studies supporting a role for MKP-1 in enhancing malignant characteristics of cancer cells, indicate that NSC 95397 is a viable therapeutic intervention for colon cancer via the inhibition of MKP-1 activity.

## Supplementary Materials

Supplementary materials can be found at http://www.mdpi.com/1422-0067/19/6/1625/s1.

## Abbreviations

MKP-1	Mitogen-activated protein kinase phosphatase-1
MEK	Mitogen-activated protein kinase kinase
ERK	Extracellular signal-regulated kinase
MAPK	Mitogen-activated protein kinase
JNK	c-Jun N-terminal kinase
ANOVA	Analysis of variance
BrdU	Bromodeoxyuridine
CDK	Cyclin-dependent kinase
Rb	Retinoblastoma protein
7-AAD	7-aminoactinomycin D
PARP	Poly(ADP-ribose) polymerase
DUSP	Dual-specificity phosphatase
IKK	IκB kinase
MKK7	Mitogen-activated protein kinase kinase 7
TBK1	TANK-binding kinase 1
NADPH	Nicotinamide adenine dinucleotide phosphate
NQO1	NADPH-quinone oxidoreductase-1
