# Supplementary material for: NSC 95397 Suppresses Proliferation and Induces Apoptosis in Colon Cancer Cells through MKP-1 and the ERK1/2 Pathway
Source: Int J Mol Sci. 2018 May 31;19(6):1625. doi: 10.3390/ijms19061625 (PMC6032145; doi:10.3390/ijms19061625)
Supplement: Supplementary file 1 [file ijms-19-01625-s001.pdf]

Supplementary Materials

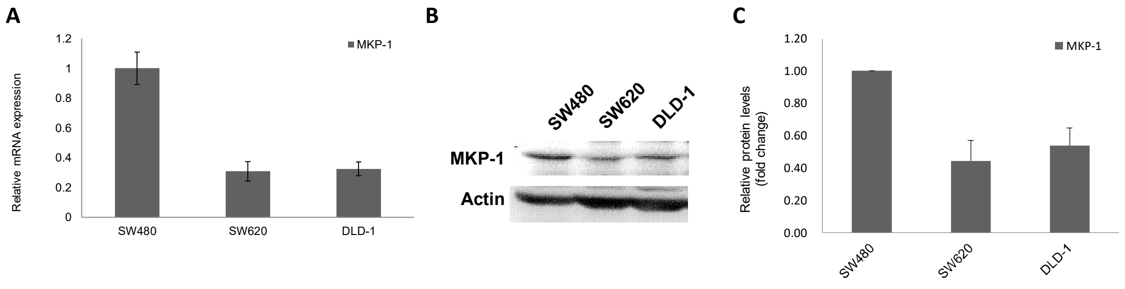

Figure S1. The expression of MKP-1 in colon cancer cells. (A) mRNA expression of MKP-1 were evaluated by real-time PCR. Values (means ± SD) indicate relative mRNA levels compared to SW480 (=1.0) after normalization to eEF1α loading control. (B) Representative western blots showing expression of MKP-1 in SW480, SW620 and DLD-1 cells, with actin as loading control. (C) Quantitative analysis of the relative protein expression of MKP-1. Values (means + SD) are normalized to actin loading and are relative to SW480 levels (=1.0).

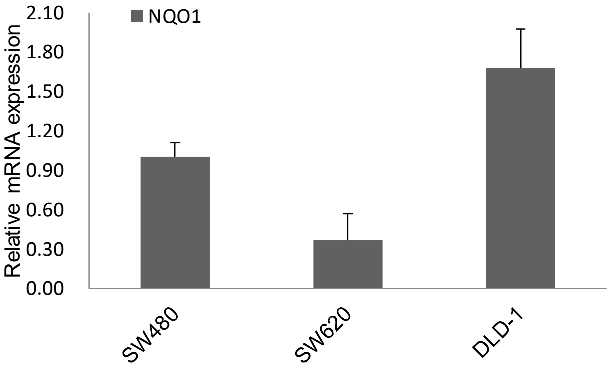

Figure S2. The mRNA expression of NQO1 in colon cancer cells. mRNA expression of NQO1 were evaluated by real-time PCR. Values (means + SD) indicate relative mRNA levels compared to SW480 (=1.0) after normalization to eEF1α loading control.
